# Cyto-3D-print to attach mitotic cells

**DOI:** 10.1007/s10616-015-9917-2

**Published:** 2015-10-13

**Authors:** Michelle R. Castroagudin, Yujia Zhai, Zhi Li, Michael G. Marnell, Joseph S. Glavy

**Affiliations:** 1 Department of Chemistry, Chemical Biology and Biomedical Engineering, Hoboken, NJ 07030 USA; 2 Warren G. Wells BioRobotics Laboratory, Stevens Institute of Technology, Hoboken, NJ 07030 USA

**Keywords:** Cytospin, Mitosis, 3D printing, Centrifugation

## Abstract

The Cyto-3D-print is an adapter that adds cytospin capability to a standard centrifuge. Like standard cytospinning, Cyto-3D-print increases the surface attachment of mitotic cells while giving a higher degree of adaptability to other slide chambers than available commercial devices. The use of Cyto-3D-print is cost effective, safe, and applicable to many slide designs. It is durable enough for repeated use and made of biodegradable materials for environment-friendly disposal.

**Electronic supplementary material:**

The online version of this article (doi:10.1007/s10616-015-9917-2) contains supplementary material, which is available to authorized users.

## Introduction

Cytospin is a cytology method that is specifically designed to concentrate and attach cells for eventual immunochemistry evaluation (Brown et al. 1996; Campaner et al. 2005; Choi and Anderson 1979; Martinazzi 1971; Shanholtzer et al. 1982; Watson 1966). Cytofunnel devices and specialized cytocentrifuges are sometimes used to concentrate cell suspension onto slide surfaces (Nassar et al. 2003; Piaton et al. 2004). In our studies on mitotic cells, we encountered difficulty in cell attachment even with adherent HeLa cells. During mitosis, cells gain a defined geometry and sufficient space for a mitotic spindle with proper orientation and correct chromosome segregation (Brown et al. 1996; Fischer-Friedrich et al. 2014). Adherent cells round up, forming spheres, this is a sign of healthy dividing cells, but the change in attached surface area leads to frequent loss of cells during experiments (Fischer-Friedrich et al. 2014). Cytospinning is employed and increases the number of mitotic cells that remain on the slide surface. Centrifuge adapters are available for standard six-well plates but not for microslide chambers (25 × 75 mm) or 35-mm slide plates. So we developed Cyto-3D-print inserts made of polylactic acid (PLA) plastic, that fit into existing centrifuge buckets for cytospinning these smaller slide chambers. Our aim in this study is to prove that Cyto-3D-print provides a rapid, safe and cost effective way to attach mitotic cells and suspension cell lines to slide surfaces.

## Materials and methods

### Mammalian cell culture

HeLa cells (ATCC, Manassas, VA, CCL-2) were grown in DMEM (Corning, Corning, NY, USA, 10-017-CV) with 10 % Fetal Bovine Serum (GE Healthcare Life Sciences, HyClone Laboratorie, Logan, Utah, USA, SH30071.03), 1 % Penicillin and Streptomycin (Life Technologies, Grand Island NY, USA, 15240-062). These monolayer cells were sub-cultured every 3–4 days.

### Reagents

Thymidine and nocodazole were purchased from Millipore (Billerica, MA, USA; Calbiochem #6060 and #487928) For immunofluorescence preparation, we utilized poly-l-lysine (Sigma, St. Louis, MO, USA; P4832), 10 % formaldehyde (Polysciences, Warrington, PA, USA; 04018) and BSA (Sigma, A3059) in Lab-Tek microslide chambers (Thermo Scientific, USA, Nunc 155361).

### Antibodies

MAb414 (Covance, Princeton, NJ, USA; MMS-120P) and anti-lamin B1 (Santa Cruz Biotechnology polyclonal gAb, Santa Cruz, CA, USA; 6216) antibodies were used for immunofluorescence with DAPI (Sigma, 471224) to visualize DNA.

### Synchronizing HeLa cells

Cells were synchronized by double thymidine and nocodazole block (Glavy et al. 2007; Kaur et al. 2010). A 2 mM concentration of thymidine was added to the medium for 18 h in which HeLa cells were grown in culture. Introducing thymidine prohibits synthesis of DNA by a negative feedback mechanism. Cells in this population go through the cell cycle and will be halted at interphase. Cells were washed 3X with phosphate buffered saline pH 7.4 (PBS) and released into the medium without thymidine for the next 8 h. Cells were then treated again with 2 mM thymidine for 18 h and released into the medium with 0.02 μg/mL nocodazole for 12 h. Cells were collected by mitotic-shake off.

### Cyto-3D-print

**Fig. 1:**
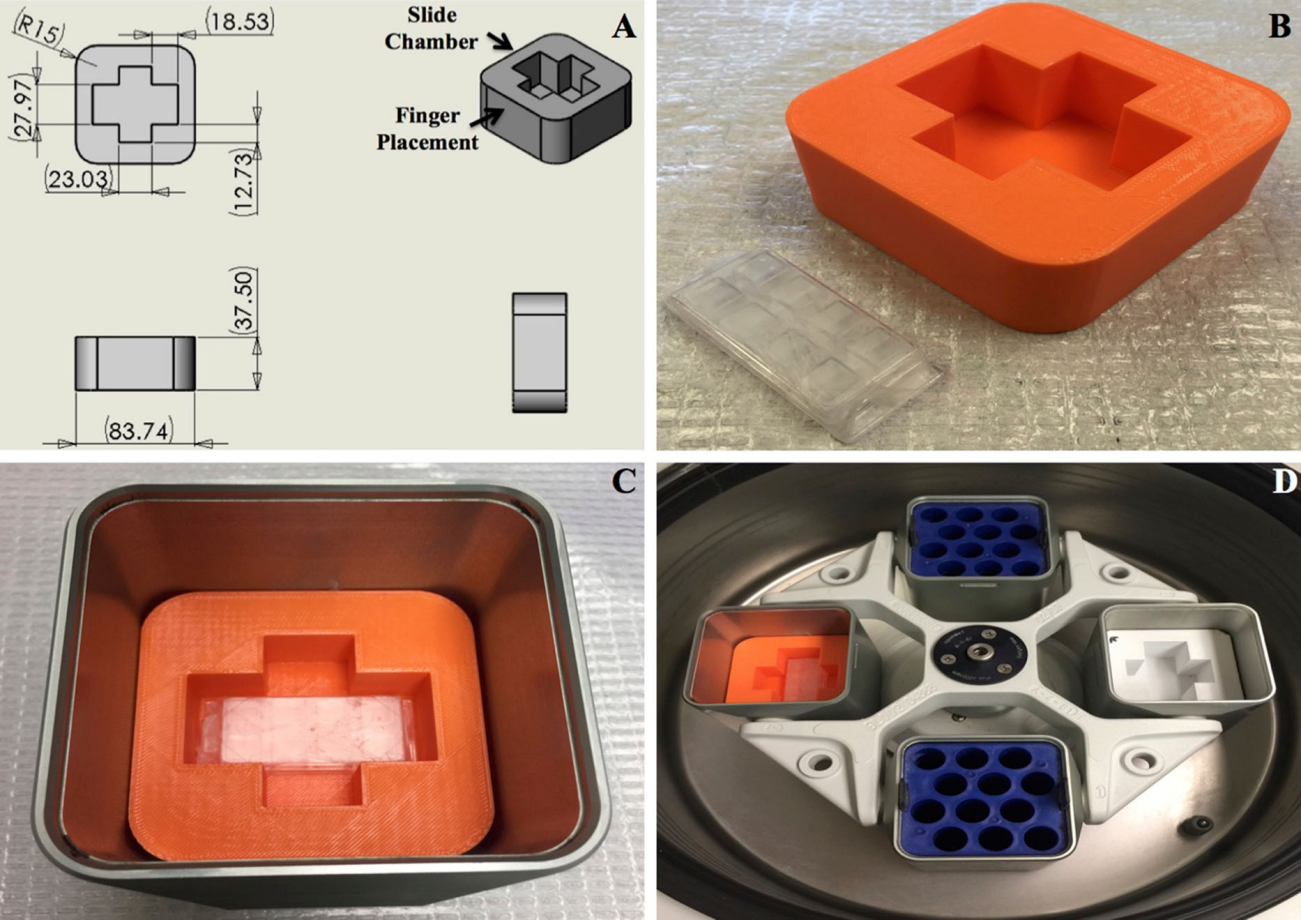
Cyto-3D-print development. *Panel A* is the solidworks 3-D print prototype designed to hold a parafilm wrapped microslide chamber within its slide chamber holder and fit inside an Eppendorf 5810R centrifuge bucket. *Panel B* is a PLA Cyto-3D-print next to a wrapped microslide chamber. *Panel C* is the Cyto-3D-print containing parafilm wrapped microslide chamber in Eppendorf 5810R centrifuge bucket. *Panel D* shows balanced device in the centrifuge rotor

3D print material used was polylactic acid (PLA) 0.22 kg Small Spool 1.75 mm PLA Filament F with Replicator Mini 3D Printer (True Orange Item: IM1UX7688 (Staples, FL, USA, $18.00) Makerbot, Brooklyn, NY, USA; Model: MP05787). We designed a prototype file in Solidworks (Dassault Systèmes, Vélizy, France) with dimensions to fit into a centrifuge bucket from an Eppendorf 5810R Centrifuge (Fig. 1a). This prototype file can be modified to fit within any centrifuge bucket as well as adjusted to fit other slide chambers inside its holder chamber. The file was designed to fit a parafilm wrapped microslide chamber across and leave enough finger-friendly space for easy placement and removal as shown in Fig. 1. A MakerBot Replicator 2x printer was used to print a plastic 3D version of our file (estimated cost under $5 per unit). Files can be printed on any 3D printer or 3D print service at very low cost ($20 for two units).

### Evaluation of cell attachment using Cytospin with the Cyto-3D-print

Cells were placed in microslide chambers, centrifuged and measured for cell attachment with and without poly-l-lysine coated slides (Glavy et al. 2007; Kaur et al. 2010).

An 8-well microslide chamber was used and set up as follows:

**Table:** 

Both poly-l-lysine and cytospinning	Poly-l-lysine only	Cytospinning only	Neither
Both poly-l-lysine and cytospinning	Poly-l-lysine only	Cytospinning only	Neither

The first and second columns were coated with poly-l-lysine a few hours before the experiment. Mitotic HeLa cells or suspension cells were first placed on the first and third columns. 10 μL of the cell suspension were placed in a hemocytometer to count how many cells were originally in each well. To ultimately test the function of the Cyto-3D-print, equal numbers of isolated mitotic cells were seeded in each well (approximately 5 × 10^4^ HeLa cells). The cells were then spun in an Eppendorf 5810R Centrifuge for 3 min at 800 rpm (130 g) (Fig. 1). The microslide chamber fits inside the Cyto-3D-print, which fits inside the centrifuge bucket (Fig. 1). The centrifuge was balanced. After, mitotic HeLa cells or suspension cells were then placed in the second and fourth columns. After a 10-min incubation period at 37 °C, 5 % CO_2_, the cells were washed twice with 250 μL of PBS. Cells were counted and photographed under a light compound microscope. The percentage of cells that remained attached to the surface chamber over the original cell number for each well was calculated.

### Immunofluorescence

Mitotic cell suspension was seeded and cyto-spun on pre-treated microslide chambers as described above. Cells were then fixed with 2 % formaldehyde in PBS for 30 min. Chambers were washed three times for 7 min with PBS and permeabilized with 0.2 % Triton X-100 for 5 min. After two additional PBS washings, cells were blocked with 5 % BSA in PBS for 1 h. Cells were washed with PBS three times (7 min each time), and incubated with designated dilutions of primary antibodies in 2 % BSA for 1 h. After PBS washing, cells were incubated with Alexa Fluor 488 or Alexa Fluor 555-conjugated secondary antibodies for 40 min (anti-mouse A21422, anti-goat A21467, Invitrogen, Carlsbad, CA, USA), which were previously diluted 1:100 in 2 % BSA-PBS. Based on the need of specific purposes, the incubation was repeated with a different pair of primary and corresponding secondary antibody. The nuclei in the cells were stained with DAPI. The prepared samples were visualized using a Zeiss LSM 5 Pascal confocal microscope.

## Results and discussion

**Fig. 2:**
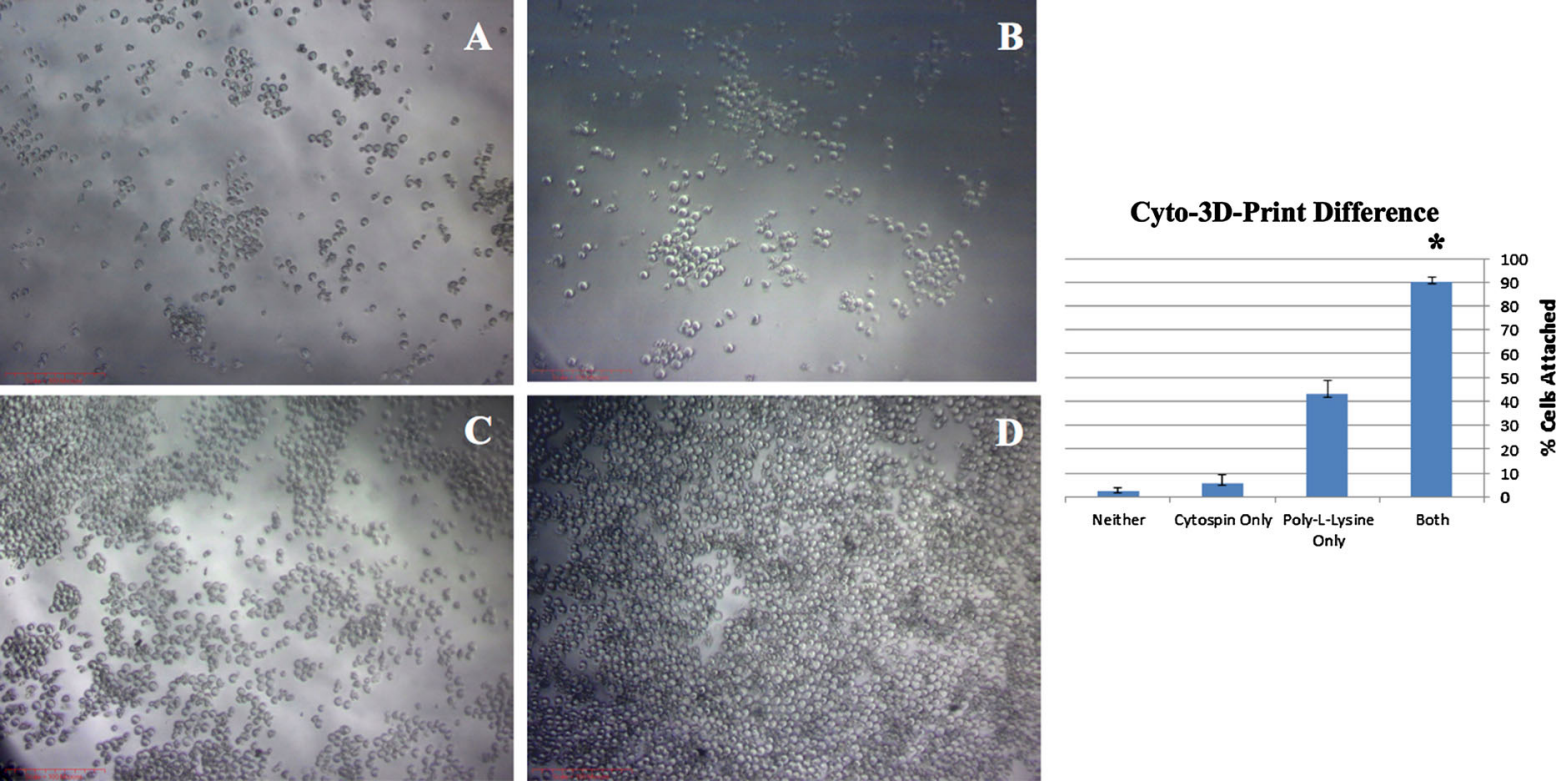
Cyto-3D-print difference: Mitotic cells were placed in a microslide chamber and measured for cell attachment with and without poly-l-lysine coating after centrifugation. In *Panel A*, just cells alone; *Panel B*, cells cytospun in Cyto-3D-print; *Panel C*, cells with only poly-l-lysine coating; *Panel D*, cells with poly-l-lysine coating and then cytospun in Cyto-3D-print. *Bar chart* shows the percentage of cells that attached to chamber surfaces with * marking the highest value

**Fig. 3:**
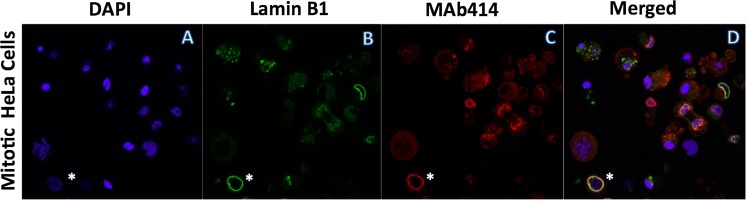
Immunofluorescence microscopy analysis of mitotic HeLa cells with nuclear envelope rim-staining antibodies against Lamin B1 and MAb414. *Panel A*: DAPI stained DNA of cells at different phases of mitosis from anaphase to telophase have all attached to the surface. *Panel B* and *C*: nuclear envelope (NE) staining by αLamin B1 antibodies (*green*) and MAb414 staining (*red*) of the nuclear pore complexes. Mitotic cells show different levels of NE staining from partial to none. *Panel D*: merged signals showing the range of mitotic cells and one interphase (*asterik*) in the *left* corner with full NE rim staining and non-condensed DNA

We have employed a simple application of plastic 3D prints for our experiments (Del Junco et al. 2015; Wong and Pfahnl 2014). Our aim is to overcome the problem of mitotic cell adherence to glass or plastic surfaces without expensive adaptors. Our results are clear and have been repeated several times with microslide chambers (Fig. 1). The plastic used was PLA from MakerBot (Nakagaito et al. 2009), which is a more biodegradable polymer compared to petroleum-based plastics. It holds its shape and is very sturdy compared to other plastics (Fig. 1b with microslide chamber). As envisioned, our Cyto-3D-print fits snuggly inside the centrifuge bucket without any gaps (Fig. 1c). In our procedure, we applied equal numbers of isolated mitotic cells in each well of the microslide chamber (Fig. 1d) and centrifuged. After, wells were washed twice with PBS then observed under a light microscope (Fig. 2a–d) and attached cells were counted for each condition (Fig. 2, bar chart of cell attachment). We found the combination of our Cyto-3D-print with poly-l-lysine coated slides had nearly 90 % attachment of the total cells added to each well, twice as good as coating alone (Fig. 2d). We observed no cracking or leakage in the parafilm wrapped microslide chambers. Our inserts are reusable and are more environmentally safe to discard than other metal based materials. Using conditions of Fig. 2d, Cyto-3D-print with poly-l-lysine coating, immunofluorescence shows the full range of mitotic cell attachment (Fig. 3). Condensed mitotic DNA is seen through the field of cells ranging from anaphase to telophase (Fig. 3a). Lamin B1 and MAb 414 mark only one interphase cell with full NE rim-staining (*, bottom left corner of panels B–D). Mitotic cells show different levels of NE rim-staining from partial to none signifying the breakdown of NE during mitosis.

In summary, our Cyto-3D-print offers an inexpensive flexible alternative to expensive metal plate adaptors with additional inserts. The use of microslide chambers has increased in recent years. Their reduced surface area saves on antibodies and reagents compared to larger six-well plates. We have developed Cyto-3D-print to bridge the gap between cytospinning and new slide technology. We hope this work stimulates more ideas to apply 3D printing to lab use.

## Electronic supplementary material

Below is the link to the electronic supplementary material.

Supplementary material 1 (MOV 166666 kb)
